# Recycling of waste ceramic foams as fine aggregates in pervious concrete

**DOI:** 10.1039/c9ra09070c

**Published:** 2020-01-13

**Authors:** Congcong Jiang, Xin Cheng

**Affiliations:** Shandong Provincial Key Laboratory of Preparation and Measurement of Building Materials, University of Jinan No. 336 Nanxinzhuang West Road Jinan 250022 China ujn_chengxin@163.com

## Abstract

The recycling of waste ceramic foams as fine aggregates to prepare a high strength pervious concrete (HSPC) was investigated. The as-obtained pervious concrete that was made using the waste ceramic foams presented promising results, with a high compressive strength of 36.54 MPa at 28 d and a favorable permeability of 3.8 mm s^−1^ compared to the traditional pervious concrete (TPC) without fine aggregates. Therefore, the dual objectives of improving the mechanical properties of pervious concrete with favorable permeability and reusing solid waste were achieved.

## Abstract 2

The recycling of waste ceramic foams as fine aggregates to prepare a high strength pervious concrete (HSPC) was investigated.

## Graphical abstract

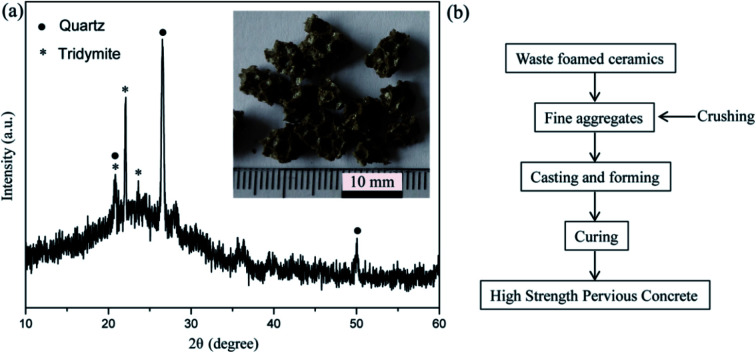

## 1. Introduction

Ceramic foams have become popular porous materials owing to the combination of being lightweight, their incombustibility, heat and sound insulating, as well as being chemically stable.^1–5^ These properties have contributed to their increased attention in the extensive production and application of ceramic foam in the fields of thermal and acoustic insulation,^6^ in light construction,^7^ as an artificial floating island carrier^8^ and for microwave absorption^9,10^ over the past ten years. While vigorously developing the ceramic foam, waste accumulation has progressively formed from discarded ceramic foam, leading to serious impacts on the surrounding environment.

Pervious concrete is obtained from coarse aggregates and minor paste with little or no sand.^11^ All the coarse aggregates are wrapped in paste and are then bonded together to form an entity with interconnected macro-voids.^12^ Therefore, pervious concrete can feature strong permeation and effective noise absorption.^13,14^ These exceptional advantages make pervious concrete one of the promising materials for sustainable infrastructure applications, including in sidewalks, alleys, plazas, gardens and in parking lots.^15,16^ However, pervious concrete with a porosity of 10–35% presents an unsatisfactory strength (28 d compressive strength hardly exceeds 35 MPa), and thus a high strength pervious concrete with acceptable permeability remains a tough challenge. Although extensive work has been done to improve the strength, most of the previous research has focused on the concrete porosity,^17,18^ the water to cement ratio,^19^ paste characteristics,^20^ the content and size of the coarse aggregates,^21^ and few studies exist that explore the role of fine aggregates, which are especially important for the structure and strength of pervious concrete.

The purpose of this paper is thus to carry out an innovative study to increase the compressive strength without causing deteriorating porosity or permeability. To this end, discarded ceramic foam was crushed and used as fine aggregates for making a high strength pervious concrete (HSPC). For comparison, traditional pervious concrete (TPC) without fine aggregates was prepared as well. The compressive strength, porosity and permeability of both samples were characterized and were analyzed in detail.

## 2. Methodology

### 2.1 Materials

**Fig. 1:**
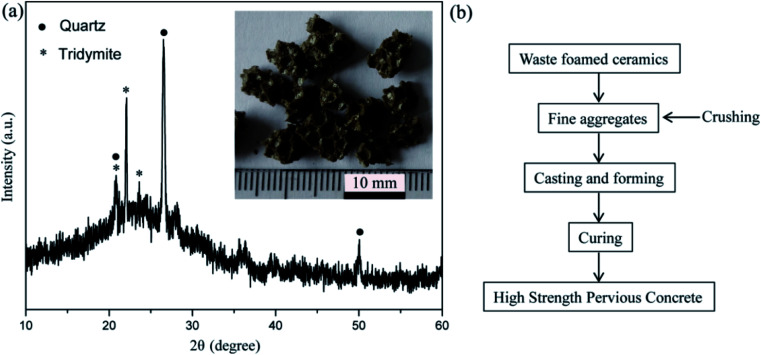
(a) XRD pattern of RFA and its image in the inset, and (b) a flow sheet of the preparation process of HSPC.

The as-received ordinary Portland cement (OPC) 42.5 R was used. Natural coarse aggregates (NCA) with an average size of 5–10 mm, a specific gravity of 2810 kg m^−3^ and water absorption of 0.2% were employed. The crushed waste ceramic foams with a particle size of 0.5–4 mm were selected as the recycled fine aggregates (RFA), as shown in the inset of Fig. 1a. The RFA were composed of quartz (SiO_2_, PDF No. 46-1045) and tridymite (SiO_2_, PDF No. 42-1401), as seen from the XRD pattern in Fig. 1a.

### 2.2 Specimen preparation

The preparation process of the HSPC sample is described in Fig. 1b. The OPC, water, NCA and RFA were added into a mortar mixer and were then mixed at a speed of 50 rpm for 5 min. In this study, a water to cement ratio of 0.41, an NCA/cement ratio of 3.85, and an RFA/cement ratio of 2.15 were selected. Then, the fresh paste was poured into cubic molds with a size of 150 × 150 × 150 mm^3^. Subsequently, the concrete specimens were stored with a relative humidity of 95 ± 5% at 20 ± 2 °C. After 24 h, the samples were removed from the molds and were then cured for 28 days.

### 2.3 Measurements of the pervious concrete

The porosity of the pervious concrete specimen was tested *via* the cold-water saturation method, according to ASTM C642-2006. The water permeability was measured by the constant hydraulic testing method, based on CJJT135-2009. The compressive strength of the samples was obtained, according to GB/T 50081-2002. A mean and standard deviation were obtained from the five measurements, to ensure the accuracy of the reported results.

## 3. Results and discussion

**Fig. 2:**
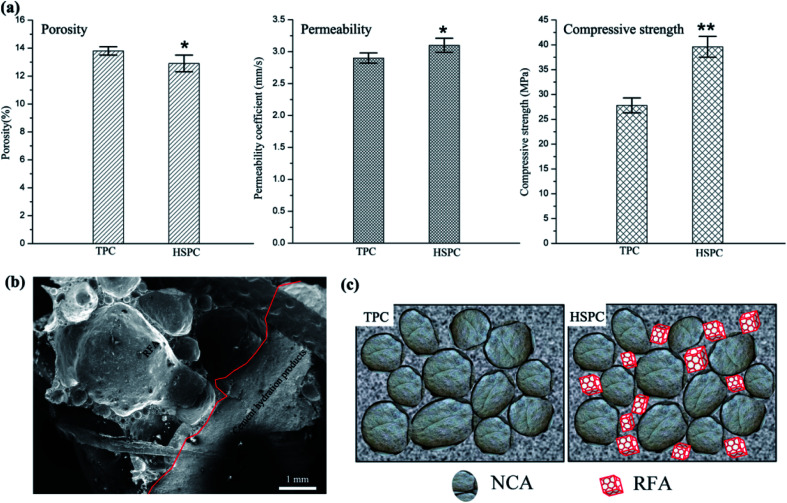
(a) Porosity, permeability and compressive strength of TPC and HSPC. Data are presented as the mean ± SD, (*) *p* < 0.05, (**) *p* < 0.01, compared with TPC; (b) cross-section of partial HSPC including RFA and hydrated cement; (c) schematic illustrations of TPC and HSPC.

Fig. 2a depicts the comparison of the porosity, permeability and compressive strength of the TPC and HSPC specimens. It can be observed that the compressive property of the HSPC specimen was 39.6 MPa, and this presents a much higher value than that of the TPC specimen (27.8 MPa). The addition of RFA resulted in the porosity of the pervious concrete ranging from 13.8% to 12.9%, whereas the permeability coefficient varied from 2.9 mm s^−1^ to 3.1 mm s^−1^. Thus, the incorporation of discarded ceramic foam as a fine aggregate did not affect the porosity and permeability much, but it produced a significant increase in the compressive strength. Such a result is exciting, because the as-obtained HSPC had more desirable permeability even at a much lower porosity in comparison to TPC, and this could be due to the structural change as a result of the incorporation of RFA. Meanwhile, the waste ceramic foams exhibited porcelain quality with high strength after firing at a high temperature,^22,23^ thus contributing to the improvement of the whole strength of HSPC.

Fig. 2b displays the cross-section of the partial HSPC including RFA and the hydrated cement. It can be observed that there were some large pores with different sizes in the RFA sample, and a large amount of small pores on the walls between the large pores was observed as well. As such, HSPC presented an innovative porous structure with multi-level pores, arising from the arrangement of NCA and NCA, NCA and RFA, as well as RFA and RFA. To conceptualize this, the structural sketches of TPC and HSPC are illustrated in Fig. 2c. TPC is a mixture of hydrated cement paste, NCA and single pores between the NCA and NCA, without fine aggregates, and thus it usually presents unsatisfactory strength. HSPC, however, incorporated with waste ceramic foam, was composed of hydrated cement paste, NCA, RCA, and multi-level pores between the NCA and NCA, the NCA and RFA, and the RFA and RFA, as well as internal pores in RFA. It is worth noting that the strength of HSPC was enhanced by the discarded ceramic foams with porcelain quality, due to the strong perturbation of the RFA arrangement. At the same time, the innovative porous structure inside the HSPC was favorable for permeability.

Recent reports on the recycling of solid wastes instead of natural coarse aggregates in pervious concrete have been documented. For example, Ngohpok *et al.*^14^ prepared pervious concrete that displayed favorable mechanical strength, accompanied with ample thermal- and sound-absorption, by replacing natural coarse aggregates with recycled concrete and coal bottom ash. Meanwhile, Yeih *et al.*^16^ investigated that pervious concrete with coarse aggregates of electric furnace slag showed desirable mechanical and permeable properties compared to that made with gravels.

In contrast to the previous reports, the typical compressive strength of conventional pervious concrete with porosities between 15% and 30% ranges from 7 to 25 MPa,^24^ and various strategies have been employed by researchers aiming to improve the strength of pervious concrete, including the construction of physical interconnected pores for prefabricated reactive powder concrete,^25^ the optimization of aggregate type and size,^26^ air-cooling electric arc furnace slag as aggregates instead of natural river gravels,^27^ the use of polymeric composites^28^ and an ultra-high performance matrix,^29^ as well as the incorporation of silica fume and ultra-fine silica powder,^28^*etc.* It is worth noting that the possibility of recycling discarded ceramic foams as fine aggregates and producing a pervious concrete with high strength and favorable permeability has been achieved, however the porosity of the pervious concrete has remained underdeveloped. Despite this, such a pervious concrete with a multi-level porous structure stands a good chance in finding broad prospects for applications.

## 4. Conclusions

High strength pervious concrete (HSPC) was fabricated by recycling discarded ceramic foam as fine aggregates. It has been demonstrated that the as-obtained HSPC presented promising results with a high compressive strength of 36.54 MPa at 28 d and favorable permeability of 3.8 mm s^−1^ compared to the traditional pervious concrete without fine aggregates, achieving the dual objectives of improving the mechanical properties of pervious concrete with favorable permeability whilst reusing solid wastes. In contrast to previous reports on pervious concrete, the possibility of recycling waste ceramic foams and producing a pervious concrete with high strength and favorable permeability has been presented, and further research regarding the effect of type and size of waste foam ceramics, as well as the durability of HSPC, will be carried out in the future.

## Conflicts of interest

There are no conflicts to declare.

## Supplementary Material
